# Social and cultural influences on genetic screening programme acceptability: A mixed‐methods study of the views of adults, carriers, and family members living with thalassemia in the UK

**DOI:** 10.1002/jgc4.1231

**Published:** 2020-03-01

**Authors:** Felicity K. Boardman, Corinna Clark, Elsita Jungkurth, Philip J. Young

**Affiliations:** ^1^ Division of Health Sciences Warwick Medical School University of Warwick Coventry UK; ^2^ School of Life Sciences University of Warwick Coventry UK

**Keywords:** attitudes, beliefs, carrier testing, decision‐making, disability, ethics, family, genetic counseling, lived experience, newborn screening, preconception, risk perception, stigma, United Kingdom

## Abstract

As population‐level carrier screening panels for reprogenetic information emerge globally, conditions to be included, and the timing of implementation is widely debated. Thalassemia is the only condition for which population‐based prenatal carrier screening is offered in the UK. However, little is known about the views and experiences of the UK thalassemia‐affected community toward this screening or other forms of genetic screening for thalassemia (newborn, preconception), despite the range of direct consequences of screening programmes for this group.

Using a mixed‐methods integrative analysis (qualitative interviews *n* = 20 and quantitative survey *n* = 80), this study outlines the experiences and attitudes of adults with thalassemia, their family members, and screen‐identified thalassemia carriers toward preconception, prenatal, and newborn screening for thalassemia. The majority of participants described thalassemia as a burdensome condition with a range of negative impacts, which contributed to their strong support for screening in all its potential formats. However, the data also highlight the challenges of each screening mode for this group, reflected in the high level of value conflict in participants' accounts and decisions. Cultural, social, and (to a lesser extent) religious factors were found to mitigate against the advantages of early screens, particularly within faith communities. Social stigma emerged as key to this process, informing the way that thalassemia severity was not only perceived, but also experienced by affected adults, which ultimately influenced screening uptake and outcomes.

These findings suggest that cultural and social sensitivity is as important as the mode of screening delivery itself, if the iatrogenic and unintended harms of screening—particularly the social/psychological burden of value conflict—are to be adequately addressed and minimized.

## 1 INTRODUCTION

With the expansion and increasing integration of genomic medicine (e.g., whole‐genome sequencing (WGS)) into state‐funded healthcare programmes (e.g., the NHS), trials of population‐wide carrier screening programmes are beginning to emerge internationally (e.g., MacKenzie's Mission, Australian Genomics Health Alliance, 2018). These programmes are designed to identify carriers of genetic conditions either before conception or during pregnancy, on the premise that information about genetic risk will expand the limited reproductive options of carrier couples. However, given the swathe of data that WGS generates, the question of which genetic variants make suitable candidates for carrier screening is widely debated (Chokoshvili, Janssens, Vears, & Borry, 2016), as well as the best timing for delivery of this information (Delatycki et al., 2019).

Thalassemia has long been considered a prime candidate for preconception genetic screening (PCGS) (Trager‐Synodinos & Harteveld, 2017) and is currently the only genetic condition for which universal prenatal genetic screening is available in the UK. Thalassemia is one of the most common inherited single‐gene disorders globally, with an estimated 270 million carriers worldwide. Mediterranean countries, Southeast Asia, India, Africa, Central America, and the Middle East, have the highest prevalence, with carrier frequencies estimated to be as high as 1:7 to 1:20 (Cousens, Gaff, Metcalfe, & Delatycki, 2010). Carrier couples have a 1:4 chance of having an affected child with each pregnancy. Efforts to reduce the incidence of thalassemia have included the implementation of mandatory premarital screening programmes (e.g., Cyprus, Iran, Saudi Arabia), with varying success rates (Cousens et al., 2010). However, approximately 7,000 infants are born with the condition each year (Al Sabbah et al., 2017).

### 1.1 Thalassemia aetiology

Thalassemia is an autosomal recessively inherited early‐onset blood disorder that results in the under‐production of hemoglobin (used by red blood cells to transport oxygen), causing severe anemia and a lifetime reliance on blood transfusions. People with thalassemia typically also require regular chelation therapy to remove excess iron from their bodies. Different types of thalassemia exist, depending on the hemoglobin gene affected (alpha/beta) and severity, with beta‐thalassemia major being the most common, but also one of the most severe, types. Thalassemia is invariably fatal without treatment, but affected individuals with regular access to medicine are now described as living into their 50s, 60s, and even 70s, suggesting that thalassemia is no longer the severely life‐limiting condition it once was (Vitrano et al., 2017). Moreover, the development of hematopoietic stem cell transplantation means that some people with beta‐thalassemia are now being described as cured of the condition, although the widespread use of this therapy remains hampered by a lack of suitable donors and therapy complications (Srivastava & Shaji, 2017).

### 1.2 Thalassemia in the UK

In England, beta‐thalassemia major is thought to affect around 1,000 people, with approximately 20–25 babies born with the condition every year (Public Health England, 2018). An estimated 300,000 people are carriers of thalassemia. Thalassemia major commonly affects people of Cypriot, Indian, Pakistani, Bangladeshi, and Chinese origin; in the UK, 80% of babies born with thalassemia have parents of Indian, Pakistani, or Bangladeshi ancestry (University College London, 2019).

Population‐level carrier screening for thalassemia during early pregnancy has been available in England since 2007 (Hewison et al., 2007). Pregnant women are offered a blood test as part of routine antenatal checks, and if found to be a thalassemia carrier, genetic testing of the father is offered. In instances of a carrier couple being identified, a diagnostic test of the fetus is offered. While uptake of diagnostic testing is relatively high, termination rates in England remain notably lower than for other prenatally diagnosed conditions: Only 40% of fetuses with a genotype diagnostic for beta‐thalassemia were terminated between 2016 and 2017, compared to 90% of those diagnosed with Down's syndrome (PHE, 2018). Newborn screening (heel prick test) is another route for diagnosis: Although thalassemia is not formally included on the newborn blood spot screen routinely undertaken, it is occasionally discovered incidentally. Carrier couples identified through prenatal screening, but for whom prenatal diagnosis is not possible/acceptable can access newborn diagnostic testing to determine the status of their child.

### 1.3 Attitudes of thalassemia‐affected individuals and families to screening

Despite thalassemia being one of the most commonly screened‐for conditions internationally (Cousens et al., 2010), few studies have explored the views of thalassemia‐affected families and screen‐identified carriers. Most studies have focused on the views of pregnant women from the general population (Tsianakas, Atkin, Calnan, Dormandy, & Marteau, 2012) and/or at‐risk ethnic minority groups (Ahmed, Green, & Hewison, 2006; Atkin, Ahmed, Hewison, & Green, 2008; Darr et al., 2013; Hanprasertpong et al., 2018), to whom thalassemia screening is targetted. Nevertheless, the accounts of affected families, adults, and carriers offer important insights into the daily realities of life with thalassemia as well as the experience of receiving, and responding, to screen‐positive results. As a such, the perspectives of this group are highly relevant to the wider population undergoing thalassemia screening.

Studies that have explored attitudes and experiences among families living with thalassemia and thalassemia carriers (e.g., Ahmed, Ahmed, Sharif, Sheridan, & Taylor, 2012; Arif, Fayyaz, & Hamid, 2008; Cousens, Gaff, Metcalfe, & Delatycki, 2013; Ishaq, Abid, Kokab, Akhtar, & Mahmood, 2012; Moudi & Miri‐Moghaddam, 2017; Ngim, Lai, Ibrahim, & Ratnasingam, 2013) have produced a mixed picture, some reporting strong support for screening (Gilani et al., 2007), and others demonstrating resistance, most frequently on grounds of religious or moral objections to pregnancy termination that may follow thalassemia identification (Atkin et al., 2008; Ishaq et al., 2012).

No studies thus far have incorporated the views of people with thalassemia themselves into the screening literature, despite growing evidence that having a screened‐for condition oneself dramatically impacts reproductive attitudes (Boardman & Hale, 2018; 2019). The literature is therefore lacking the perspectives of those with the most intimate and realistic knowledge of what life with thalassemia is like, insight of great relevance to the design of screening programmes, and those screened.

In response to this gap in the literature, this study examines the views of adults with thalassemia, family members of people with thalassemia, and screen‐identified thalassemia carriers toward different types of screening programme for the condition. Attitudes to screening and the social and cultural context within which these reprogenetic decisions are grounded are examined through the use of mixed data analysis (20 qualitative interviews and 80 quantitative survey responses). By outlining the social, ethical, and cultural challenges to screening implementation, this study contributes to the wider debate regarding the timing, suitability, and applications of whole‐genome sequencing as a population screening tool.

## 2 MATERIALS AND METHODS

An exploratory mixed‐methods sequential design was adopted (Creswell & Plano Clark, 2006), using three distinct phases, as set out below. Firstly, exploratory interviews were conducted with adults with thalassemia, family members and screen‐identified carriers between June and December 2017. The resulting analysis of these interviews was then used to inform the development of a national survey, which was implemented between January and June 2018. The quantitative analysis of the survey data was conducted separately at first, before being integrated with the phase one qualitative interviews, through a process of mutual illumination and direct integration techniques (Kaur, Vedel, Sherif, & Pluye, 2019). The initial qualitative themes developed in phase one were then revised to incorporate the findings of this mixed‐methods analysis. Ethical approval for the study was granted by the Biomedical and Scientific Research Ethics Committee (University of Warwick) and by the NHS Ethics Committee and Health Research Authority (IRAS ID: 226508).

### 2.1 Phase 1: Qualitative interviews and survey development

Qualitative interviews were initially conducted with 20 people living with thalassemia in some capacity. Participants were included if they were aged over 18, able to communicate in English and either had thalassemia themselves, had the condition in the family, or were a carrier (with or without family history). Calls for interview participants were advertised through the UK Thalassaemia Society and within a large NHS regional genetics center in England. Twenty‐five people responded to these advertisements; however, two responders were excluded as they did not have experience with thalassemia, and attempts to set up interviews with a further three responders were unsuccessful despite repeated efforts.

Twenty participants took part in in‐depth interviews (see Table 1 for a breakdown of characteristics). The interviews explored participants' experiences with thalassemia and their attitudes toward reprogenetic technologies that identify carriers and diagnose fetuses/newborns with thalassemia, as well as their perceived uses of that information.

**Table 1:** Qualitative interview participants

Participant pseudonym	Age	Gender	Ethnicity	Religious faith	Affected children/total children	Relationship to thalassemia	How thalassemia discovered
Yazeed	52	M	Asian (Pakistani)	Muslim	0/5	AwTh, FM	Symptoms
Rima	25	F	Asian (Lebanese)	Eastern Orthodox	0	AwTh	Symptoms
Ameena	38	F	Asian (British Indian)	Muslim	1/4	SiTC, FM	Screening
Giovanni	36	M	European (Italian)	Catholic	0	AwTh, FM	Symptoms
Farrah	33	F	Asian (Pakistani)	Muslim	3/3	SiTC, FM	Screening
Sa'id	45	M	Asian (Iraqi)	Muslim	0/5	AwTh	Symptoms
Nasarin	25	F	Asian (Iranian)	Muslim	0	AwTh, FM	Symptoms
Safina	36	F	Asian (Bangladeshi)	Muslim	0	AwTh	Symptoms
Deeba	49	F	Asian (Pakistani)	Muslim	1/2 (adopted)	FM	Child's symptoms
Aarav	68	M	Asian (British Indian)	Hindu	1/1	SiTC, FM	Child's symptoms
Jamini	52	M	Asian (British Indian)	Hindu	1/2	SiT, FM	Child's symptoms
Zania	44	F	European (Sardinian)	Catholic	0	AwTh	Symptoms
Maryam	35	F	European (Greek Cypriot)	Greek Orthodox	0	AwTh	Symptoms
Afra	40	F	Asian (Pakistani)	Muslim	1/2	SiTC, FM	Newborn screening
Arjun	41	M	Asian (British Pakistani)	Muslim	1/4	SiTC, FM	Screening
Daania	36	F	Asian (Afghani)	Muslim	0/0	SiTC	Screening
Talia	35	F	European (Italian/Maltese)	Catholic	0/1	SiTC, FM	Screening
Mishel	21	F	Asian (Armenian)	Muslim	0/0	SiTC	Screening
Sophie	29	F	White British	Christian	0/0	SiTC	Screening
Xanthe	34	F	European (Greek Cypriot)	Greek Orthodox	0/0	SiTC	Screening

Abbreviations: AwTh, adult with thalassemia; FM, family member; SiTC, screen‐identified thalassemia carrier.

FB, an experienced qualitative researcher, was responsible for all interviews, and an interview guide was used to inform their content. A separate interview guide was prepared for participants with thalassemia and their families (see Appendix S1), and thalassemia carriers identified through antenatal screening (see Appendix S2), due to the contrasting levels of familiarity with the condition, although both guides covered the same topic areas.

Fifteen interviews were conducted over the telephone and five face‐to‐face, depending on participant preference. The five face‐to‐face interviews took place either in participants' homes (3) or in a hospital clinic (2). Interviews lasted between 40 and 70 min, were audio‐recorded, and transcribed verbatim. Transcripts were returned to all 20 participants to give them the opportunity to check/amend the resulting text, although in practice only one participant took up this offer.

The analysis was conducted using a modified grounded theory approach to ensure the resulting themes were data‐driven (Glaser & Strauss, 1967). An iterative process of theme identification and refinement, with the aid of Nvivo software (v.11), was conducted until theoretical saturation had occurred.

Following completion of this analysis, the core themes were used to develop a survey instrument, the Thalassaemia Screening Survey (UK) (see Appendix S3), with verbatim text from the interviews used directly in combination with Likert scales where possible. The survey was designed to measure attitudes toward two different models of thalassemia screening currently unavailable in England: PCGS and NGS. Demographic questions were replicated (or modified) from the 2011 UK Census.

### 2.2 Phase 2: National survey and quantitative analysis

Survey data collection took place between January and June 2018. Inclusion criteria were as follows: Over 18, UK resident, diagnosed with thalassemia, known to be a thalassemia carrier, or has thalassemia within the family. No restrictions were placed on the nature of the familial relationship as experience with the condition was prioritized over biological relationship (i.e., step, adopted, and fostered family members were all eligible to be included).

A paper version of the survey was mailed to 500 households known to the UK Thalassaemia Society, and a link to an online version was distributed through their online networks, as well as the social media accounts associated with the research project. Participants were encouraged to distribute the survey to eligible family/friends. Postal returns were processed using data scanning technology to reduce inputting error. Eighty surveys were returned and included in the quantitative analysis.

Due to the small numbers of participants in some subcategories, Fisher's exact tests were used to compare responses between groups, for example, by religious faith. Responses were stratified into agree (agree and strongly agree), disagree (disagree and strongly disagree), and other (don't know, neither agree or disagree, and prefer not to say). Missing data were excluded from analyses.

### 2.3 Phase 3: Mixed‐methods data analysis

In the final stage of analysis, the qualitative interview data were revisited, using direct and indirect mixed‐methods integration techniques (Creswell & Plano Clark, 2006) to compare and aid understanding of the quantitative findings. This mixed analysis resulted in refined qualitative themes where the survey data provided additional or contrary insights than those generated by the qualitative analysis alone. The findings reported in this paper are derived from this final analysis and are structured around the qualitative themes that emerged from the mixed integration. Quotations were selected that most eloquently represented the key theme being presented.

## 3 RESULTS

The results of this paper are organized to reflect phase three of the analysis strategy. Demographic information is firstly presented before moving on to a presentation of the final core overarching themes.

### 3.1 Demographic information

#### 3.1.1 Qualitative data

The qualitative dataset comprised of 8 people with thalassemia (3 were also family members as they had affected siblings), 11 carriers who had been identified through antenatal screening (7 of whom had affected children), and 1 family member (a parent) who had adopted a child with thalassemia (Table 1).

The affected adults, adoptive parent, and screen‐identified carrier parents (*n* = 16) were all recruited through the UK Thalassaemia Society, while the four screen‐identified carriers (without affected children) were recruited through an NHS regional genetics center. Of the 15 participants who knew the form of thalassemia in their family, 13 (90%) were affected by beta‐thalassemia major and two by thalassemia intermedia. Interviewees were mostly female (70%), and all reported belonging to a religious faith. The majority (11; 55%) were Muslim, seven (35%) belonged to a denomination of Christianity (Catholic, Church of England, and Greek/Eastern Orthodox), and two (10%) were Hindu.

#### 3.1.2 Quantitative data

Of the 80 people who completed the survey, 23% were family members and 76% had thalassemia. Eighty‐five per cent had experience with beta‐thalassemia major, and 63% were female (Table 2). The majority of the sample (77%) reported that they belonged to a religious faith, with a similar spread to the qualitative sample; the largest group of religious participants was Muslim (35% of the whole sample), followed by Christian (28%) and Hindu (14%). Forty‐seven participants in the survey (59% of the sample) were of reproductive age (defined as 18–45 years of age), and of those of reproductive age, the majority were Muslim (24 participants; 51%). In contrast, the majority of the Christian (64%) and Hindu (55%) participants fell into the older age brackets (45–66+) (Table 2).

**Table 2:** Survey participants demographics by religious faith

	Total *n* (%)	Religious faith *n* (%)				Fisher's exact test*
		Christian	Muslim	Hindu	Other	
Gender						
Female	51 (64)	20 (71)	16 (57)	5 (45)	10 (77)	NS
Male	27 (34)	7 (25)	12 (43)	6 (55)	2 (15)	
Not specified	2 (3)	1 (4)	(0)	(0)	1 (8)	
Age (years)						
18–25	6 (8)	1 (4)	3 (11)	1 (9)	1 (8)	*p* = .027
26–34	11 (14)	1 (4)	7 (25)	1 (9)	2 (15)	
35–45	30 (38)	8 (29)	14 (50)	3 (27)	5 (38)	
46–55	20 (25)	11 (39)	2 (7)	3 (27)	4 (31)	
56–65	9 (11)	6 (21)	1 (4)	1 (9)	1 (8)	
66+	3 (4)	1 (4)	0 (0)	2 (18)	0(0)	
Not Specified	1 (1)	0(0)	1 (4)	0(0)	0(0)	
Ethnicity						
Asian/Arab	43 (54)	2 (5)	26 (60)	11 (26)	4 (9)	*p* < .001
White European	33 (41)	24 (73)	1 (3)	0 (0)	8 (24)	
Other/not specified	4 (5)	2 (50)	1 (25)	0 (0)	1 (25)	
Educated to degree level and above						
Yes	39 (49)	11 (39)	14 (50)	7 (64)	7 (54)	NS
Children						
Yes	21 (26)	8 (29)	9 (32)	3 (27)	1 (8)	NS
Relationship with thalassemia						
Family member	18 (23)	8 (29)	6 (21)	4 (36)	0 (0)	NS
Has thalassemia	61 (76)	20 (71)	21 (75)	7 (64)	13 (100)	
Not specified	1 (1)	0 (0)	1 (4)	0 (0)	0 (0)	
Type of thalassemia						
Alpha	2 (3)	1 (4)	0 (0)	1 (9)	0 (0)	NS
Beta intermedia	6 (8)	1 (4)	3 (11)	0 (0)	2 (15)	
Beta major	68 (84)	23 (82)	24 (86)	10 (91)	11 (85)	
Other/don't know	4 (5)	3 (11)	1 (4)	0 (0)	0 (0)	
Current health and well‐being						
Good/very good	45 (63)	20 (80)	11 (44)	8 (80)	6 (55)	*p* = .039
Fair/bad/very bad	26 (37)	5 (20)	14 (56)	2 (20)	5 (45)	
Total	80	28 (35)	28 (35)	11 (14)	13 (16)	

* NS = not significant, ‘not specified’ categories excluded from analysis.

### 3.2 Key themes

#### 3.2.1 Living with thalassemia: Severity and stigma

Perceptions of life with thalassemia within both datasets were somewhat negative, with 63% of survey participants believing that living with thalassemia necessarily causes suffering (Table 3). Nevertheless, 83% of the entire survey sample still reported that a good quality of life can be achieved with the condition (Table 3). Further detail about the lived realities of life with thalassemia was obtained from the qualitative dataset; all but one of the participants with thalassemia (7/8) emphasized that while they had managed to achieve important life goals, they experienced significant periods of illness, restriction, and reduced quality of life. Restrictions affected many spheres of life, most commonly employment, education, travel, finances, fertility, and relationships, were a source of frustration and disappointment and significantly impacted on self‐image. Zania, diagnosed with thalassemia at the age of two in Sardinia, described life with the condition as follows:

The problem is the lack of freedom. Well, you have a freedom, but's always on a leash kind of because yes I can do a lot, but I cannot do as much as other people can, I'll soon need another transfusion, and that gives you a lack of self confidence in your body…[…]… at the age of 18 I wanted go to university and the only thing that stopped me is that it was outside Sardinia and my mum didn't allow me to go there because she thought I couldn't cope alone… (Zania, 44, has thalassemia)

**Table 3:** Attitudes toward thalassemia and preconception screening by religious faith

	Response	Religious Faith *n* (%)				All *n* (%)	Fisher's exact test (*p*)*
		Christian	Muslim	Hindu	Other/none		
Attitudes to thalassemia							
Having thalassemia causes people to suffer	Agree	15 (54)	20 (71)	6 (55)	9 (69)	50 (63)	**.029**
	Disagree	2 (7)	7 (25)	3 (27)	3 (23)	15 (19)	
	Other^a^	10 (37)	1 (4)	2 (18)	1 (8)	14 (18)	
People with thalassemia and their families are well supported by society	Agree	6 (21)	10 (36)	1 (9)	2 (15)	19 (24)	**.014**
	Disagree	13 (46)	16 (57)	9 (82)	4 (31)	42 (53)	
	Other	9 (32)	2 (7)	1 (9)	7 (54)	19 (24)	
People with thalassemia can have a good quality of life	Agree	24 (86)	23 (82)	9 (82)	10 (83)	66 (84)	.772
	Disagree	1 (4)	3 (11)	2 (18)	1 (8)	7 (9)	
	Other	3 (11)	2 (7)	0 (0)	1 (8)	6 (8)	
Quality of life with thalassemia varies greatly depending on severity	Agree	23 (82)	25 (89)	6 (60)	12 (92)	66 (84)	.160
	Disagree	2 (7)	0 (0)	2 (20)	1 (8)	5 (6)	
	Other	3 (11)	3 (11)	2 (20)	0 (0)	8 (10)	
Attitudes toward preconception screening							
I would support a preconception genetic screening programme for thalassemia	Agree	26 (93)	24 (89)	11 (100)	12 (92)	73 (92)	.973
	Disagree	0 (0)	1 (4)	0 (0)	0 (0)	1 (1)	
	Other	2 (7)	2 (7)	0 (0)	1 (8)	5 (6)	
Identifying carriers of thalassemia in the general population will lead to carriers feeling stigmatized or different	Agree	4 (14)	13 (48)	4 (40)	3 (23)	24 (31)	**.023**
	Disagree	18 (64)	8 (30)	4 (40)	3 (23)	33 (42)	
	Other	6 (21)	6 (22)	2 (20)	7 (54)	21 (27)	
People from the general population won't be interested in finding out their carrier status as they won't think it's relevant to them	Agree	6 (21)	15 (56)	8 (73)	8 (62)	37 (47)	**.003**
	Disagree	14 (50)	10 (37)	1 (9)	1 (8)	26 (33)	
	Other	8 (29)	2 (7)	2 (18)	4 (31)	16 (20)	
It will be harder for thalassemia carriers to get married and/or have children once their genetic status is known about	Agree	8 (29)	13 (48)	6 (60)	2 (15)	29 (37)	**.017**
	Disagree	16 (57)	11 (41)	4 (40)	4 (31)	35 (45)	
	Other	4 (14)	3 (11)	0 (0)	7 (54)	14 (18)	
Identifying carriers of T before a pregnancy is conceived will affect people's choice of reproductive partner (the person you choose to have a baby with)	Agree	14 (52)	20 (74)	4 (40)	10 (77)	48 (62)	.131
	Disagree	8 (30)	4 (15)	3 (30)	0 (0)	15 (20)	
	Other	5 (19)	3 (11)	3 (30)	3 (23)	14 (18)	
	Total	28 (35)	28 (35)	11 (14)	13 (16)	80	

a Other response = neither or don't know.

* Significant differences by faith group in bold.

While the impacts of thalassemia itself were a key part of Zania's difficulties *‘I cannot do as much as other people…[..]…I'll soon need another transfusion’*, the role of wider society in shaping the experience of life with thalassemia was woven throughout the qualitative and quantitative data. Indeed, only a minority of survey participants (24%) thought that people with thalassemia and their families were well supported by society (Table 3) and a lack of awareness among the general population was also noted; 47% of survey participants thought that preconception screening for thalassemia carriers would be considered of little relevance by the general population (Table 3). For many of the interviewees as well, this combination of societal ignorance and the unavailability of social support were hallmark features of the stigma they experienced. Sa'id, for example, reported a life punctuated by judgment, exclusion, and rejection:

… but I have been judged all my life. Teachers made judgements on me that I couldn't do O‐levels because I was always in hospital, and when I did get a job I really, really worked hard even though I was feeling ill all the time. But they used my sickness, you know, going into hospital, against me and took me out of promotions…so it's not easy to get a job with thalassaemia, you know, because they're judging me on it…even though the government say it's all fair and straight, it's not, because the people at the end of the day will see that you've ticked disability and then it goes onto a separate pile, you don't hear back from them at all. And it's the same with dating, as soon as they know about it, they move on. You're always on the rubbish heap. (Sa'id, 45, has thalassemia, 5 unaffected children)

All eight interview participants with thalassemia reported that their lives were overshadowed by the social stigma of having a genetic disease. For Sa'id, the impact of being ‘judged’ for having thalassemia was as damaging to his overall well‐being as the physical impact of the condition itself: ‘*I think you can have a perfectly fine life with thalassaemia actually if you get all the support you need, it's just how the community respond to it’*, suggesting that the intersection of genetic disease with society and culture impacts not only how others view the condition, but also transforms into the physical and psychological realities of those with the condition.

Despite stigmatization of genetic conditions and carrier status being widely reported during interviews, only 31% of the survey participants reported perceived stigma around thalassemia carrier status, although this varied significantly by religious faith; a greater proportion of Muslims, compared to Christians, associated thalassemia carrier status with stigma (48% vs. 14% respectively, Table 3). In addition, 37% of survey participants agreed that carrier status could cause difficulties for carriers wanting to get married, and the majority of the whole survey sample (62%) thought that carrier identification would affect people's choice of reproductive partners, which strongly suggests the stigmatizing consequences of carrier status, particularly among minority groups (Table 3).

#### 3.2.2 Preconception screening for thalassemia: Identity, self, and relationships

Reducing thalassemia‐related terminations was reported as a priority for nearly all interviewees (19/20 spontaneously mentioned this conviction) and was considered a key benefit of preconception, over prenatal genetic screening. Interviewees generally framed this in terms of moral, pro‐life, and/or religious convictions, as Daania, a carrier, commented:

I think because I'm pro‐life, you know a Muslim, I just think that if God has given a couple the benefit of having children and if that child were to be sick, maybe that's God's will…You can't take that life, that's not your decision to make. So maybe finding out [carrier status] before a pregnancy is…better because then you won't be tempted to make that choice, to take that from God's hands. (Daania, 36, thalassemia carrier, pregnant)

Like Daania, most believed that identifying carriers before pregnancy might prevent the difficult choice between having a ‘sick’ child and ending a pregnancy. However, the route by which this termination prevention would occur was not clear, as for the vast majority of qualitative and quantitative participants, a change in reproductive partner was not considered a likely outcome of preconception screening. Indeed, only one interviewee (Maryam) suggested the use of pre‐implantation genetic diagnosis (PGD) (embryo screening), a potential option for those carrier couples identified before conception. Maryam, diagnosed with thalassemia intermedia at 18 months old, described the difficulties of abandoning an established relationship with her Italian partner, Davide, after he was found to be a carrier shortly before their marriage:

We were just shocked really….Obviously I knew that the chances were…because I think one in seven people from Italy are [carriers]…I had wanted him to get tested earlier on, but he kept saying ‘there's nothing wrong with me’. So…in hindsight…We probably would have never got together…We talked seriously about separating, because we knew…that the road to having children…was no longer going to be a smooth one, and maybe this was God's way of saying we shouldn't be together. But yeah it's not that easy when you've been together a long time… So yeah we didn't [separate] in the end, although our families both felt it might be best. (Maryam, 35, has thalassemia)

Given Maryam's strong moral and religious objections to pregnancy termination, and the higher than usual chances of their children inheriting thalassemia (1 in 2), she and Davide went on to use one round of PGD, although this was ultimately unsuccessful.

While Maryam supported preconception screening, her view that it was still ‘*too late in the day*’ was also raised by two other people with thalassemia, who felt that earlier screens (e.g., adolescence), would prevent the trauma and stigma of separation after a significant amount of time and emotion had been invested in a relationship. However, ignorance of the meaning of carrier status and concerns about self‐identity was raised as obstacles to such earlier screening. For example, Davide initially resisted undergoing carrier screening, as he perceived that being a carrier would imply something ‘wrong’ with his own health. Some interviewees were skeptical about how seriously the information would be taken, particularly given the low level of knowledge about thalassemia among unaffected populations ‘…*the point at which you really need the information [*about carrier status*] is when you're young and you just don't think about that stuff when you're young, you think you're invincible*’ (Yazeed, 52, has thalassemia with 5 unaffected children). However, participants suggested various routes for community education, including schools, religious groups, television, and social media, which they felt could lead to more informed reproductive decisions.

There was a significant difference between religious faiths concerning attitudes to marriage for carriers. Twenty‐nine per cent of the Christian participants thought that it would be difficult for a carrier to marry, compared to 48% of Muslim and 60% of Hindu participants (Table 3). The qualitative data confirmed this finding, particularly among Muslim participants, suggesting that knowledge of carrier status (and associated stigma) at an earlier time point could influence marriage negotiations, and 13/15 interview participants expressed concern about their own, or their children's, future marriages in light of genetic information. As Daania, commented:

…..there is just a lot of stigma attached within the South Asian community about people that have any sort of medical conditions really….so they just think ‘oh she's got a problem, let's not have to deal with that issue’. So I'd be expected to sort of notify a potential partner and their family about it prior to a marriage arrangement, they'd have to get tested…and it just creates a lot of issues, and if there're problems they wouldn't have to deal with with a non‐thalassaemia family, then me and my family get passed over. (Daania, 36, thalassemia carrier, pregnant)

This sense of social stigma also led some participants with thalassemia and their families to actively conceal the condition, as highlighted by Rima, a 25‐year‐old woman with thalassemia:

….it was interesting when I was growing up, we kept thalassaemia in the immediate family, so between my parents, me and my brother, we didn't talk about it to outsiders. I think extended family members knew, but it was never outwardly acknowledged. I think for my parents, they never wanted it to become a ‘thing’.… they were conscious about what the reaction might be towards me….if I were to suddenly be treated differently just because of this. (Rima, 25, has thalassemia)

While Rima did not refer to any specific examples of ‘enacted stigma’ during her interview, a sense of ‘anticipated’ stigma played a significant role in the way in which she and her family managed day‐to‐day life with thalassemia (Earnshaw, Quinn, & Park, 2011).

#### 3.2.3 Prenatal screening for thalassemia: Experience and the moral minefield

While participants generally supported prenatal screening, citing ‘choice’ and ‘information’ as the key drivers for support, the interviews also highlighted the inherent difficulties involved in discovering an inherited genetic condition during pregnancy, particularly in the context of this highly religious population. Talia, a practising Catholic, was 35 and pregnant with her first child when she discovered her carrier status during routine antenatal screening. She later discovered that other members of her family were also carriers of thalassemia:

I was about 13 weeks [pregnant] and I just remember getting this phone call from this lady and it all sounded very serious and they said they'd found a thalassaemia trait, and I'd have to come and get my husband tested. They scared me half to death. And actually it all made sense, as I'd had some health issues with anaemia previously and I found out from my mum that there were some cases in the family….so we did some research into the family history about the trait, and went and spoke to relatives, about what it meant for me and possibly also baby, because it was important for me to hear it from them when it's part of our family tree. And so yeah I felt prepared then….But…it probably wasn't the best time to give me that news in many respects, because it was all quite urgent and stressful when really it could have waited until after Matthias was born, but I guess they say fore‐warned is fore‐armed. (Talia, 35, thalassemia carrier, 1 unaffected child)

While Talia's husband was not a carrier, Talia's religious convictions meant that her prenatal screening result was viewed primarily as health risk information, rather than as relevant to reproductive behaviors. Her previous experiences with anemia enabled Talia to incorporate thalassemia into her own and her family's health narrative, using the collective experiential knowledge of the family as preparation for Matthias' birth.

However, for other screen‐identified carriers, a lack of family history and ignorance of the condition were important influences on the way in which carrier results were interpreted. Farrah and her husband, parents to three children with thalassemia, were informed that they were a carrier couple during Farrah's first pregnancy, but assigned little significance it:

…..at the time we got pregnant and yeah they said we were carriers…..I just didn't realise how severe it was or how it would affect me or my child. I just fobbed it off like ‘oh you know, they say a lot of things'….1 in 4 didn't seem too bad, I thought well that's three chances it won't happen…. and I didn't take it seriously because I'd just never heard of it, and if it was serious I would have heard of it, wouldn't I? (Farrah, 33, thalassemia carrier with three children with thalassemia)

While Ayra was diagnosed with thalassemia shortly after birth, which Farrah described as *‘devastating’,* Ayra initially appeared to be mildly affected and did not require blood transfusions until she was four years old. These experiences led Farrah to describe thalassemia as *‘not that bad really’*, a factor in their decision to proceed with a second pregnancy without intervention. However, this child, Sajid, was also diagnosed with thalassemia at birth and required transfusions by 12 months of age. By her third pregnancy, with two children undergoing thalassemia treatment and the associated impact on family life, Farrah's sense of genetic risk had dramatically altered. While previously dismissing invasive prenatal diagnostic tests, she now felt a competing sense of genetic responsibility and the weight of perceived expectations from healthcare professionals:

I prepared myself, went for the counselling, actually went on the day to [hospital], got there and I just changed my mind because I was three months gone and I just thought, I can't do it, because what if it is healthy and I end up losing it, or what if it has it [thalassaemia] and I am expected to terminate? So I was like in so many different minds that I couldn't carry on. And then when Ranya was born and they told me she had it, I was really upset again…. but either way I wouldn't have got rid of any of my children, Islamically I'm not allowed to anyway‐ it would be a big crime. (Farrah, 33, thalassemia carrier with 3 children with thalassemia)

For Farrah, religious objections to termination as a ‘*big crime’* (along with fears of pressures to undergo one) rendered the miscarriage risk associated with prenatal testing unacceptable; her belief ultimately overriding her fears of having a third child with thalassemia and enabling her to present her decision both responsible and moral, despite the counterpressure she anticipated.

#### 3.2.4 Newborn screening for thalassemia: Gender and responsibility

Support for newborn screening for thalassemia was very high among survey participants (95%) (Table 4) and slightly higher than was reported for preconception screening (92%) (Table 3). The overwhelming majority of survey participants (95%) agreed that newborn screening was a means by which to improve the health care and support offered to affected families, with 90% agreeing that a diagnosis of thalassemia within the neonatal period is still useful, even if, at that stage, disease severity cannot be accurately determined. Indeed, the frequent critique of newborn screening—that a diagnosis at such an early (and likely presymptomatic stage) might interfere with the early bonding between parent and infant—was not strongly expressed in this surveyed group (Table 4).

**Table 4:** Attitudes toward newborn screening by religious faith

	Response	Religious faith *n* (%)				All *n* (%)	Fisher's exact test (*p*)
		Christian	Muslim	Hindu	Other/none		
I would support a newborn genetic screening programme for thalassemia	Agree	27 (100)	23 (85)	11 (100)	13 (100)	74 (95)	**.049**
	Disagree	0 (0)	0 (0)	0 (0)	0 (0)	0 (0)	
	Other^a^	0 (0)	4 (15)	0 (0)	0 (0)	4 (5)	
Identifying thalassemia at birth would interfere with early bonding between parent and child	Agree	1 (4)	2 (8)	0 (0)	1 (8)	4 (5)	.557
	Disagree	22 (85)	17 (65)	7 (64)	9 (69)	55 (72)	
	Other	3 (12)	7 (27)	4 (36)	3 (23)	17 (22)	
Identifying thalassemia at birth would lead to better support and health care for the child and their family	Agree	26 (96)	24 (92)	11 (100)	12 (92)	73 (95)	.912
	Disagree	0 (0)	0 (0)	0 (0)	0 (0)	0 (0)	
	Other	1 (4)	2 (8)	0 (0)	1 (8)	4 (5)	
Even though parents would not know for sure how severely affected their newborn baby will be, it is still better that they know about thalassemia straight away	Agree	26 (93)	23 (85)	10 (91)	12 (92)	71 (90)	.870
	Disagree	0 (0)	0 (0)	0 (0)	0 (0)	0 (0)	
	Other	2 (7)	4 (15)	1 (9)	1 (8)	8 (10)	
Identifying thalassemia at birth is important as it would enable parents to make informed decisions about any future pregnancies	Agree	26 (93)	24 (89)	11 (100)	12 (92)	73 (92)	.795
	Disagree	1 (4)	0 (0)	0 (0)	0 (0)	1 (1)	
	Other	1 (4)	3 (11)	0 (0)	1 (8)	5 (6)	
	Total	28 (35)	28 (35)	11 (14)	13 (16)	80	

a Other response = neither or don't know.

* Significant differences by faith group in bold.

Given this positivity toward newborn screening observed within the quantitative data, it is noteworthy that the one interview participant, Afra, who had first‐hand experience of a thalassemia diagnosis in her newborn (detected as an incidental finding), was far more ambivalent about the value of this type of screening. Afra described her daughter, Jawhara's diagnosis as a *‘bolt from the blue’*, highlighting its detrimental and depleting impact on her experience of the newborn period:

I was just in shock really… and I think I was the worst off, being the mother, I brought her into the world so I was to blame. My husband was a lot more, you know, calmer about it. …It took me a long time to recover from that shock, and I became quite depressed…. post‐natal depression I guess. You just think, alright, I'm going to have a happy healthy baby, and then that suddenly crashes down and you realise this child has a problem, when you're already very tired and emotional. (Afra, 40, thalassemia carrier with 1 child with thalassemia and 1 unaffected)

For Afra, social and cultural expectations of mothers as child‐bearers—and consequently more heavily implicated in reproductive outcomes—were integral to how she responded to Jawhara's diagnosis, ultimately contributing to her postnatal depression, *‘I brought her into the world, so I was to blame’*.

Despite this sense of personal failing, Afra acknowledged that the unanticipated diagnosis nevertheless enabled her to access prenatal diagnosis in her subsequent pregnancy, a benefit most commonly associated with preconception screening, and a one that was widely acknowledged as important within the quantitative data on newborn screening (92% agreement, Table 4).

As well as a felt sense of heightened responsibility for Jawhara's diagnosis, Afra was also pivotal to the distribution of genetic risk information and policing of testing decisions within her own family:

I just think now, if you're about to get married, or when you meet someone, you should both get it checked out. Particularly if you're Asian. I made my brother and sisters also have tests to make sure they, to give it some awareness. Because if we had known we would have been prepared, we would have read up on it… we still had another child, you know, it wasn't like we were like, ‘oh my god we're never going to have any more children again!’, it wouldn't have put us off, it just would have saved us the shock at a time I should have been full of joy and enjoying my daughter. (Afra, 40, thalassemia carrier with 1 child with thalassemia and 1 unaffected)

Despite most survey participants being unconcerned about the effect on early bonding or inability to know severity immediately (Table 4), Afra reported that Jawhara's initial diagnosis of beta‐thalassemia major (later ‘down‐graded’ to thalassemia intermedia) did in fact negatively impact their early bonding:

….We were so, so lucky….because the doctors did scare us up a lot and say ‘she's got thalassaemia major……when she's a year old she's going to need transfusions’. That's what I was coming to terms with. And we were so blessed that it didn't go that way and she was intermedia, but I do regret that now. I…felt our lives were over and we grieved for that, when really, we needn't have. But you never get that time back…And I honestly found it hard to bond with her at first because I really thought she was going to die. (Afra, 40, thalassemia carrier with 1 child with thalassemia and 1 unaffected)

While Afra appreciated the value of early diagnosis for hers and her family's subsequent reproductive decisions, it came at a high personal cost, and one she was unable to anticipate.

#### 3.2.5 Negotiating screening: Resistance and deflection

The qualitative and quantitative data together demonstrate the significance of social, religious, and cultural factors in informing attitudes toward, and experiences of, thalassemia and screening. However, there was evidence that these influences were not rigid and could be challenged or deflected.

While Zania, for example, acknowledged the prevalence of social stigma around thalassemia, she was able to transform this into a positive assertion about her identity:

…..But I also think that in some way it [thalassaemia] helped me shape my personality. Because I was outside the general pack of teenagers I… yes it was difficult, but at the same time I think it gave me the impetus to be me, and very early on I decided I think, I'm going to be very much an individual who makes her own decisions, who doesn't follow societal norms, who… it gave me a lot of individualism I think. (Zania, 44, has thalassemia)

Zania's perceived difference to her peers supported her emerging sense of self‐determination, separating her from ‘societal norms’ and expectations, and enabling her to challenge and redefine restrictions. Rather than accepting a negative label, Zania emphasized the character‐building nature and unique insights gained from growing up with a condition that made her stand out from the crowd.

As well as social and cultural ideas around thalassemia, there was also evidence of participants challenging religious ideas, particularly around pregnancy termination, as Sa'id commented:

I know a lot of Muslims will probably be telling you that abortion is a sin, you have no right to do it, and yes it is, but this isn't about ending a life, it's a medical issue. You know, God gave you a brain to use and you have to apply it how you see fit, and I don't think any God would want to see this thing [thalassaemia] continue, so the right thing is to end the suffering, not continue it, but people get so indoctrinated they don't see that. (Sa'id, 45, has thalassemia with 5 unaffected children)

Unlike many, who positioned religion and medicine as diametrically opposed on pregnancy termination for thalassemia (e.g., Farrah), Sa'id demonstrated the possibility of mediating conflict through prioritizing his own moral understandings and personal responsibility. By reframing pregnancy termination as a ‘medical’ rather than ‘religious’ issue, Sa'id transcended the rigidity of religious doctrine, simultaneously retaining his commitment to his faith. Sa'id's views demonstrate the limitations of using religious affiliations as a proxy for views on pregnancy terminations and highlight the malleability of divisions between medicine and religion.

## 4 DISCUSSION

This study, to the best of our knowledge, is the first to examine the attitudes of people with thalassemia, their family members, and thalassemia carriers (with and without family history) toward population genetic screening in different formats. The findings help to explain the ambivalence, and sometimes contradictions, across and between qualitative and quantitative datasets, by demonstrating the complexity of views and experiences, the range of impacts associated with genetic screening, and the outward ripple effect of screening information to extended family and wider community.

The survey demonstrates that support for thalassemia screening is high in all formats among affected families and higher than reported among families living in the UK with another inherited blood disorder, hemophilia (Boardman, Hale, Gohel, & Young, 2019). Newborn screening garnered particularly high levels of support among participants (95%) (compared to 77% support for newborn screening among families affected by hemophilia), even though it would not reduce the prevalence of thalassemia, instead acting as a form of tertiary prevention (Belhoul, Abdulrahman, & Alraei, 2013). Instead, newborn screening was presented as important for the early provision of support for thalassemia‐affected families, the perceived lack of which, as well as awareness of thalassemia, was strongly evident throughout the qualitative and quantitative data as well as the surrounding literature (Cousens et al., 2010; Sapountzi‐Krepia et al., 2006). While survey participants did not perceive negative impacts on infant/parent bonding of an early diagnosis, a common ethical concern of newborn screens (Frankel, Pereira, & McGuire, 2016) the qualitative data suggested that there may be implications for parental guilt and blame particularly for mothers, which may have been dispersed across both partners in cases of preconception or even prenatal screening (James, Hadley, Holtzman, & Winkelstein, 2006; Hallowell et al, 2003).

Prenatal screening was broadly supported; however, for many this was considered far too late in reproductive pathways to impact pregnancy outcomes and was the screening method most highly associated with value conflict. Of the seven interviewees identified as being a carrier couple through prenatal screening, all went on to have affected children, reflecting the trend against pregnancy termination for thalassemia in the UK and beyond (PHE, 2018). This trend against pregnancy terminations among thalassemia carrier couples has often been associated with religious convictions (Al Sabbah et al., 2017; Karimi et al., 2007), although this has recently begun to be challenged (Ahmed, Atkin, Hewison, & Green, 2006; Ahmed, Green, et al., 2006; Atkin et al., 2008).

Indeed, this study confirms the suggestions of other research in this area that negative perceptions of the severity of thalassemia were far more likely to inform attitudes to pregnancy termination, over and above religious imperatives (Ahmed, Atkin, et al., 2006; Atkin et al., 2008; Shaw, 2011). Beyond this, however, this study also highlights the significant role that social and cultural stigmas play in shaping—and even *creating*—that disease severity. Both the qualitative and quantitative data reinforced previous findings that Middle Eastern cultures have highly stigmatized views of disability and health conditions (Van den Heuvel et al., 2008). Interviewees perceived reductions in opportunities for marriage, work, and reproduction—stemming from stigma—as being as damaging to a person's life chances as the physical disease itself, confirming previous research on the role of social stigma in determining life experiences of people with genetic disease (Clarke, 2016; Hoffman‐Andrews, Mazzoni, Pacione, Garland‐Thomson, & Ormond, 2019). This study therefore highlights the porous boundaries between bodily impairment and social/environmental factors in creating the experience of disability for individuals (Hughes, 2004), highlighting that social factors, such as stigma, are *integral to* the profile of genetic conditions and consequently of direct relevance to the design of carrier screening programmes in different global contexts.

In countries such Iran and Saudi Arabia, punitive social consequences, associated with unfulfilled marriage arrangements or pregnancy terminations, have a large influence on screening uptake (Alswaidi et al., 2012; Chattopadhyay, 2006; Karimi et al., 2007) and have been cited as explanations for the continuation of marriages despite both parties being identified as carriers, and the continuation of pregnancies once thalassemia is identified (AlHamdan, AlMazrou, AlSwaidi, & Choudhry, 2007; Moudi & Miri‐Moghaddam, 2017). Interview participants spoke candidly of their entire families being ‘*blacklisted*’ following the discovery of carrier status (e.g., Daania) and of people being *‘shunned’* by family and community members following a pregnancy termination.

Such competing social and ethical dilemmas collided and jostled for priority within participant's accounts of thalassemia screening (e.g., Farrah and Talia). Participants had to negotiate the contradictions between values inherent to Western medicine (e.g., an emphasis on knowledge and informed choice (Van den Heuvel & Marteau, 2008)) and the cultural norms and religious practices of their families and communities, leaving many walking a precarious tightrope between competing accountabilities. Some participants (e.g., Farrah) responded to this by strategically privileging one factor (religious objection to termination) to invalidate others (stigma of having a child with thalassemia) and to exonerate themselves from negative (and often punitive) social consequences of making this decision ‘incorrectly’, although inadvertently reinforced stigmas in the process.

While it could be argued that the existence of screening programmes, and their attendant reproductive decisions, can therefore actually *reduce* the reproductive autonomy of patients when set in the context of their particular social, cultural, and religious contexts, this study also demonstrates that responses to this interaction could also be a site of transformation. It would be inaccurate to characterize the participants in this study as passive recipients of social stigma, religion, and culture, as there was evidence of the development of strategies of resistance, creativity, and flexibility in responses to them. Zania, for example, transformed stigmatization of having a genetic disease into a positive characteristic, which demonstrated her ‘uniqueness’. Similarly, Sa'id, by drawing a distinction between religious dogma and moral reasoning, was able to define thalassemia as a ‘medical’ rather than ‘religious’ issue, reconciling the maintenance of his view that thalassemia‐affected pregnancies should be terminated.

With the proliferation of new genomic technologies such as whole‐genome sequencing, this study underscores the need for population‐level genetic screening programmes to be both culturally sensitive and socially responsive in their design and implementation. Social, religious, and cultural influences not only create moral and personal tensions (Chattopadhyay, 2006; Boardman et al, 2017), but, importantly, also shape the lived reality of the condition itself. Therefore, incorporating lived experience (including social/cultural influences), into the concept of disease severity (rather than biological factors alone), is an important first step toward a contextualized understanding of screening impacts.

Only when these factors are addressed may people whose lives are touched by the condition be appropriately supported in managing the value conflicts that emerge through screening with the least social and psychological burden possible.

### 4.1 Study limitations

The sample size for this study was relatively small, with a poor response rate from the postal and online survey. This low response rate may be due to the high degree of stigma and secrecy surrounding a thalassemia diagnosis which was evident throughout the research process. Biases may have been introduced by the recruitment of most participants through a support organization, the UK Thalassaemia Society. An NHS regional genetics center was used to counterbalance this recruitment bias, but only procured seven interview participants.

The sample achieved also reported a high degree of religiosity with 100% of interview participants and 84% of survey participants reporting belonging to a religious faith—the majority identifying as Muslims. However, when considered alongside ethnicity data from the study (95% of survey and interview participants were of South Asian or Mediterranean backgrounds), this high degree of religiosity appears consistent with that reported among these ethnic minority groups, which are the ethnic groups most commonly affected by thalassemia.

Both within the survey and interviews, female participants were over‐represented (70% of interview participants and 64% of survey participants), which may reflect a greater willingness on the part of women to discuss reproductive attitudes and behaviors than men. Indeed, women are social positioned as more heavily implicated in reproductive outcomes than men, which may have impacted the perceived relevance of the study to women's lives.

Despite these limitations, qualitative and quantitative participants nevertheless demonstrated a wide range of experiences with thalassemia which offset somewhat the limitations of recruitment, for example, carriers with/without family history, those with/without affected children, and participants with differing religious convictions and views on pregnancy termination. Perceptions of thalassemia may have been influenced by the fact that 80% of survey participants and 90% of interview participants had experience with beta‐thalassemia major, the most severe form of thalassemia, although this is nevertheless the most common form of the condition so is unlikely to reduce the relevance of the findings.

### 4.2 Practice implications

This study has a range of practice and policy implications. Firstly, genetic counselors who are likely familiar with the complexity of patient views around genetics might usefully be included in the design of population‐wide carrier screening programmes. As genomic medicine expands to encompass whole population screening, the role of the genetic counselor is similarly shifting, encompassing counseling of increasing numbers of people who lack prior knowledge of the conditions screened for, and who are likely unprepared for a positive result (Ioannou, Delatycki, Massie, Hodgson, & Lewis, 2015). Genetic counselors have much expertise and insight to bring to the development of informed consent processes within such carrier screening programmes. The contribution of personal stories and insights, or providing an advisory role within the development of patient literature, for example, may enable patients to better imagine the scenarios that may arise from participation in genetic screening programmes, within the context of their own lives, before screening is undertaken.

This study also highlights the importance of sensitivity in genetic counseling practice to the way in which social stigma may play a role, not only in the way that genetic testing decisions are approached, but also its role in creating and sustaining particular lived experiences with genetic conditions. Genetic counselors have an important role to play in alerting patients to the limitations of genetic information in accurately predicting the lived realities of future people with genetic conditions such as thalassemia in an age wherein genetic information is often interpreted as deterministic (Haga et al., 2013). To do this, genetic counselors may encourage patients to explore the full range of factors (social, physiological, cultural, environmental etc.) that combine to create life with a genetic condition, as well as their expectations and beliefs about people affected. For members of particular minority ethnic cultures and religions, social factors—notably stigma—may have a particularly prominent role in influencing perceptions of disease severity, with a range of consequences for affected families. Genetic counselors are uniquely positioned to explore the values and experiences underpinning such stigmas, practice which has a role to play in reducing the social and emotional toll of value conflict for thalassemia‐affected families and carrier couples as identified within this study.

### 4.3 Research recommendations

Further research is needed to explore drivers of and resistance to social stigma and cultural and religious factors (including gender, social class, nature of the impairment, and mode of inheritance) on the lived reality of genetic disease and reproductive decision‐making.

## AUTHOR CONTRIBUTIONS

All authors contributed to the analysis, paper writing, and final approval of the manuscript. Boardman was responsible for data collection, and Boardman, Clark, Jungkurth, and Young all contributed substantially to data analysis and integration. Boardman is in agreement to be accountable for all aspects of the work in ensuring that questions related to the accuracy or integrity of any part of the work are appropriately investigated and resolved.

## COMPLIANCE WITH ETHICAL STANDARDS

### Conflict of interest

Boardman, Clark, Jungkurth, and Young declare they have no conflicts of interest to declare.

### Human subjects and informed consent

Written informed consent for participation in the qualitative interviews and the national survey was obtained before data collection and following provision of an information leaflet on the study. No identifiable data from participants were retained, and all names appearing in this paper are pseudonyms.
