# Supplementary material for: Social and cultural influences on genetic screening programme acceptability: A mixed‐methods study of the views of adults, carriers, and family members living with thalassemia in the UK
Source: J Genet Couns. 2020 Mar 1;29(6):1026–40. doi: 10.1002/jgc4.1231 (PMC7754126; doi:10.1002/jgc4.1231)
Supplement: Supplementary file 1 [file JGC4-29-1026-s001.docx]

**Interview Schedule**

**Thalassaemia interviews: Adults with Thalassaemia and Family Members**

These questions are intended to be a loose guide and will be adapted as appropriate for each interview and condition affecting the family. Participants will also be given considerable freedom to direct the interview.

1. Can you tell me a bit about yourself? (Age/occupation/living arrangements/family/hobbies and interests)
2. How would your friends/family describe you?
3. Can you tell me your story of life with thalassaemia?

-prompt for story of:

- pre-diagnosis
- diagnosis/prognosis
- responses to diagnosis/prognosis
- reactions of family
- daily (quality of) life with condition
- which aspect(s) of the condition are the most concerning?
- has the condition prevented you/them from achieving any life goals?
- reproductive decisions since diagnosis in family
- duration of condition in family
- what support has been available for you/your family member? Is this sufficient?
- do you know anyone else with thalassaemia?
- are you or they active in the condition’s community (e.g. support group)?

1. How would you describe thalassaemia to someone who has never heard of it before? What are the important things they should know about it in order to understand what the condition is like to live with?
2. Do you think the possibility of undergoing antenatal screening for conditions like thalassaemia is a useful thing for the general public to be able to access? Why (not)?
3. What are your views on pre-conception carrier screening for thalassaemia? (screening for carrier status before a pregnancy is established)? What do you think are the pros and cons of screening for thalassaemia in this way?
4. What do you feel the general population need to understand about thalassaemia in order to make informed pre-conception genetic screening decisions?
5. How/when might you/have you, or members of your family, used prenatal or genetic testing yourself? Why (not)?
6. When do you think is the best time for people to learn that they are carriers of thalassemia, or that their child has thalassaemia? Why do you think this? (prompt for pros and cons of different screening programmes)
7. Knowing what you do, if you could be in the room when a couple are told they have a pregnancy affected by thalassaemia, what would you want to say anything to them? -prompt for discussion around different severities.
8. How/would their decision-making differ from yours if you were in that situation?
9. How do you think people from the general population view thalassemia? Do you agree?
10. Would you view screening for thalassaemia differently to screening for other conditions that are currently screened for, e.g. Down’s Syndrome? Why (not)? –prompt for views around different types of condition (physical, behavioural, visible, treatable, life shortening etc.).
11. What do you understand by the term ‘quality of life’? What do you think you need to have to have a good ‘quality of life’? Can people diagnosed with thalassaemia enjoy a good quality of life, according to your understanding of the term? Why (not)?
