# Supplementary material for: Social and cultural influences on genetic screening programme acceptability: A mixed‐methods study of the views of adults, carriers, and family members living with thalassemia in the UK
Source: J Genet Couns. 2020 Mar 1;29(6):1026–40. doi: 10.1002/jgc4.1231 (PMC7754126; doi:10.1002/jgc4.1231)
Supplement: Supplementary file 2 [file JGC4-29-1026-s002.docx]

**Thalassaemia carriers interview schedule**

1. Can you tell me a little bit about yourself (age/occupation/family etc.)
2. Can you tell me the story of how you came to have an appointment at clinical genetics?
3. Did you realise you were being screened for thalassaemia carrier status before you received your result?
4. Had you ever hear of thalassaemia before you received your result?
5. Have you met anyone with thalassaemia before?
6. What is your understanding of thalassaemia now? Can you tell me about the day you received your carrier status result: how was the result given? Were you given information that was useful? What worked well and what could have gone better?
7. Can you tell me about your very first reaction to your carrier result? Have you told anyone else about your result? If so, who?
8. Has knowledge of your carrier status influenced any decisions you have made since then? (prompt: partner testing, foetal diagnosis as/when appropriate) What does being a ‘carrier’ mean to you? (prompt: do you see yourself as a healthy person? ? If so, in what way?)
9. What relevance (if any) do you think this carrier result has for your life? (prompt: does it have any implications for others? How will you/have you used the information)
10. Do you know anyone else who is a thalassaemia carrier?
11. Do you think knowing your thalassaemia carrier status is a good thing? If so, when is the best time for people to be screened and receive this information? Do you think your thalassaemia carrier status will influence your decisions around having children in the future? Why (not)?
12. Do you view screening for thalassaemia carrier status any differently than screening for other conditions in pregnancy, such as Down’s Syndrome? How is it different?
13. Will you encourage your child to undergo thalassaemia carrier screening when they are old enough? (if relevant) What about other family members?
14. Do you think preventing conditions like thalassaemia through screens during pregnancy is a good thing? Why (not)?
15. Did you receive enough information, counselling and support from the NHS?
16. Is there anything else you want to add?
