# Supplementary material for: Social and cultural influences on genetic screening programme acceptability: A mixed‐methods study of the views of adults, carriers, and family members living with thalassemia in the UK
Source: J Genet Couns. 2020 Mar 1;29(6):1026–40. doi: 10.1002/jgc4.1231 (PMC7754126; doi:10.1002/jgc4.1231)
Supplement: Supplementary file 3 [file JGC4-29-1026-s003.docx]

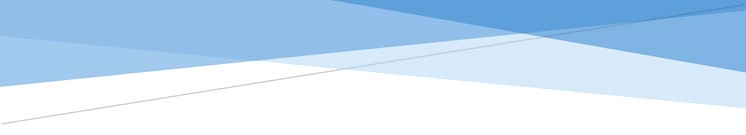


THALASSAEMIA

SCREENING SURVEY (UK)

A survey of the views of families living with thalassaemia in the UK on the possibility of pre-conception/prenatal genetic screening

Dr. Felicity Boardman

Division of Health Sciences

Warwick Medical School

# Gibbet Hill Road Coventry CV4 7AL

Felicity.Boardman@warwick.ac.uk screeningstudy@warwick.ac.uk

# 024 761 51291

www.warwick.ac.uk/imagining_futures

0338139186

**Thalassaemia Screening Survey (UK)**

Welcome to the Thalassaemia Screening Survey (UK). This survey is part of a research project called 'Pre-conception genetic screening for conditions of uncertain or variable prognosis: social and ethical implications’

(http://www.warwick.ac.uk/imagining_futures). This survey has been designed to explore the views of people living with thalassaemia (or who have a relative with the condition) towards the possibility of screening the population pre-conceptually (before a pregnancy is conceived) for thalassaemia. 'Screening' refers to the identification of thalassaemia in the whole UK population, not just within families already known to be affected by the condition. A screening programme would mean that people without a family history of thalassaemia would be offered the same opportunities to undergo pre-conception or prenatal genetic screening that are currently only offered to affected families.

Whilst the NHS Sickle Cell and Thalassaemia (SCT) screening programme, which offers genetic screening to all pregnant women and fathers-to-be (where antenatal screening shows the mother is a genetic carrier) was introduced in 2001 and all newborn babies (as part of the newborn blood spot screening programme) are screened in the UK, there is currently no newborn screening programme available to the general population that can detect thalassaemia.

**Why is the study being done?** At present, very little is known about how families currently living with genetic conditions such as thalassaemia feel about the possibility of a pre-conception or newborn genetic screening programme. This survey is designed to address this gap in understanding. The outcome of this survey will be submitted to any screening policy reviews for thalassaemia screening carried out by the UK National Screening Committee (the advisory body to government on issues related to screening). To develop this survey, 15 in-depth interviews were undertaken with people who either have thalassaemia, or have it in their family. These interviews gave us a picture of the sorts of views people living with thalassaemia have on screening, and these views have been incorporated into the survey.

**Why am I being asked to take part?** You are being invited to complete this survey if you are aged over 18, live in the UK and have thalassaemia in your family, or have it yourself. We are interested to hear from a range of family members (e.g. aunts/uncles, cousins, siblings, grand-parents, step- and half-relatives) or anyone who considers themselves a 'family member' of someone with thalassaemia, regardless of whether you are biologically related, and regardless of whether your relative with thalassaemia is still living, or has died. People, and family members of those, who have been cured of thalassaemia with a bone marrow transplant are also eligible to take part.

**How do I participate?** Simply fill in the Thalassaemia Screening Survey (UK) and return it in the pre-paid envelope, or to the address found at the back of this survey. The survey takes about 15-20 minutes to complete. Please complete the survey as an individual, rather than as a couple or family, as everyone feels differently about screening (even within couples and families) and it is important to get an accurate picture of the range of views on this topic. If you prefer, you may complete this survey online at:

http://www.warwick.ac.uk/thalassaemiascreeningsurvey (active from 21.05.2018)

0477139180

**What will happen to my information if I participate?** The anonymised summary results of this survey (not individual data) will be published in academic journals, written up as a research report for the UK Thalassaemia Society’s newsletter and also used for conference presentations (academic, professional and patient). It will also be submitted as research evidence to any policy review of thalassaemia screening conducted by the UK National Screening Committee. Upon completion of the research project, the anonymised data from this survey will be archived with the UK Data Service (https://www.ukdataservice.ac.uk/) so that in the future, other researchers may make use of the data. ***If you do not want your anonymised survey answers to be archived, please contact us by email or telephone (screeningstudy@warwick.ac.uk / 02476 151291) BEFORE completing the survey.***

**What if I change my mind?** If you start the survey and decide you do not want to continue for whatever reason, simply do not return it. However, once a completed survey is returned, it will not be possible to withdraw it from the study. This is because the survey is anonymous and therefore it will be impossible to link an individual back to their survey.

**Will my taking part be kept confidential?** All data collected from this survey will be held anonymously and securely using data encryption software. No data which may identify you (e.g. your name/address) will be asked for, but you will be asked for background information about yourself. This is in order that we can get a clearer understanding of the backgrounds of the people responding. All data will be handled in strict accordance with the Data Protection Act 1998.

**Who is conducting the research?** This research is being conducted by Dr.

Felicity Boardman (Assistant Professor) and Dr. Rachel Hale (Research Fellow), at Warwick Medical School, and is funded by the Wellcome Trust. The research was given a favourable opinion by Warwick's Biomedical and Scientific Research Ethics Committee on 5th April 2018 (REGO-2017-1910 AM04).

**Are there any disadvantages to taking part?** Some people may find the topic of screening for thalassaemia upsetting. Should the completion of this survey raise any issues for you which you would like support with, or further information on, please contact your GP or the clinician who ordinarily treats your thalassaemia.

**What if there’s a problem?** Any complaint about the way you have been dealt with during this study will be properly addressed by a person who is independent of the study. Please address your complaint to: Deputy Director/ Head of Research Governance, Research & Impact Services, University House, University of Warwick, Coventry CV4 8UW. Or email: researchgovernance@warwick.ac.uk

If you have any comments or queries about the survey, or to request further paper copies, please contact us at screeningstudy@warwick.ac.uk or complete the comments box at the end of the survey. **You may want to tear off and keep the cover page of this survey before returning it in order to retain the project details. Alternatively, email** (**screeningstudy@warwick.ac.uk**) **and ask for this information to be posted or emailed to you.**

Thank you!

8444139182

## THALASSAEMIA SCREENING SURVEY (UK)

### Section 1: About You

In this section you will be asked for basic background information about yourself. These questions are being asked in order that the researcher can gain a better understanding of the social backgrounds of people living with thalassaemia in their family.

| **1. What is your sex?** |  |
| --- | --- |
| Male | Female |
| **2. What is your age?** |  |
| 18-25 years46-55 years  26-34 years56-65 years  35-45 years66+ years | |
| **3. What is your highest qualification level?** | |

No qualifications

GCSE or O Level

GCE, A level or similar

Vocational (BTEC/NVQ/Diploma)

Degree level or above

Other (please specify):

**4**

**. What is your ethnic group?**

White- British

White- Irish

White- Gypsy or Traveller

White- European

Mixed- White and Black Caribbean

Mixed- White and Black African

Mixed- White and Asian

Asian- Indian

Asian- Pakistani

Asian- Bangladeshi

Asian- Chinese

Black- African

Black- Caribbean

Arab

Prefer not to say

Other (please specify):

**5**

**. Do you have a religious faith?**

Yes

No

Prefer not to say

Christian (any denomination)

Jewish

Muslim

Sikh

Hindu

Buddhist

Other (please specify):

**If yes, how would you describe your religious faith?**

9993139182

### Section 2: Thalassaemia: You and Your Family

In this section you will be asked questions about the number of people affected by thalassaemia in your family, any treatment you are currently receiving, as well as your perceptions of your own, and your family members' health and well-being.

**6. Do you have any children who have (or used to have) thalassaemia? (This might include step-children, foster children, adopted children or any child for whom you consider yourself to be the parent or legal guardian)**

Yes No

If yes, how many children do you have?

| **7. What is your relationship to thalassaemia? *(please tick all that apply)*** |
| --- |
| I have thalassaemia myself  Someone in my family has, or had, thalassaemia |

1. **What type of thalassaemia do you have yourself, or do you have in yourfamily? *(please tick all that apply to you and your family)***

Alpha thalassaemia major / Haemoglobin H Disease

Beta thalassaemia major

Beta thalassaemia intermedia

Delta thalassaemia

Other (please specify):

Don't know

| **8A. Have you, or your relative, to the best of your knowledge, ever received contaminated blood in your treatment for thalassaemia?** |
| --- |
| Yes No Don't know |

**8B. If yes, please state which conditions were contracted as a result of receiving this contaminated blood (please tick all that apply):**

HIV

Hepatitis C

Another condition (please specify):

4178139182

1. **Please list your relationships to family members (to a maximum of 10) who are or has been affected by thalassaemia. This may include extended family members. If your family member(s) have died, please sate their approximate year of death and age at death (if known), e.g. Grandmother, died 1990, aged 80. If no one else in your family has or had thalassaemia, please go on to question 11.**

|  | **Your relationship to family members with THALASSAEMIA, their age and date of death (where applicable), e.g. son, died 1990 aged 14** |
| --- | --- |
| **a.** Family member 1 |  |
| **b.** Family member 2 |  |
| **c.** Family member 3 |  |
| **d.** Family member 4 |  |
| **e.** Family member 5 |  |
| **f.** Family member 6 |  |
| **g.** Family member 7 |  |
| **h.** Family member 8 |  |
| **i.** Family member 9 |  |
| **j.** Family member 10 |  |

1. **Do you currently, or have you at any point in the past, lived in the samehousehold as your family member(s) listed in question 9 (please answer for each family member as listed in the order above)? ‘Living in the same household’ would include living together on a temporary basis with your family members affected by thalassaemia, e.g. regular holidays or stays of more than two weeks in the same household.**

|  | **Currently, or at some point in the past, lived in the same household?** | |
| --- | --- | --- |
|  | **Yes** | **No** |
| **a.** Family member 1 |  |  |
| **b.** Family member 2 |  |  |

0353139183

|  | | **Currently, or at some point in the past, lived in the same household?** | | | | | | | | |
| --- | --- | --- | --- | --- | --- | --- | --- | --- | --- | --- |
|  |  | **Yes** | | | | **No** | | | | |
| **c.** Family member 3 | |  | | | |  | | | | |
| **d.** Family member 4 | |  | | | |  | | | | |
| **e.** Family member 5 | |  | | | |  | | | | |
| **f.** Family member 6 | |  | | | |  | | | | |
| **g.** Family member 7 | |  | | | |  | | | | |
| **h.** Family member 8 | |  | | | |  | | | | |
| **i.** Family member 9 | |  | | | |  | | | | |
| **j.** Family member 10 | |  | | | |  | | | | |
| **11. How would you rate your current health and well-being?** | | | | | | | | | | |
| Very good Good Fair Bad Very Bad | | | | | | | | | | |
| **12. How would you rate the current health and well-being of your family member(s) currently or previously affected by thalassaemia listed in question 9? Please rate in the order you listed them in above. If you have no family members currently or previously affected by thalassaemia, please go on to Section 3.** | | | | | | | | | | |
|  | | **Current Health and Well-being of Family Members with THALASSAEMIA** | | | | | | | | |
|  |  | **Very good** | | **Good** | **Fair** | **Bad** | | **Very Bad** | **Don't know** | **Not applicable**  (family member died) |
| **a.** Family member 1 | |  | |  |  |  | |  |  |  |
| **b.** Family member 2 | |  | |  |  |  | |  |  |  |
| **c.** Family member 3 | |  | |  |  |  | |  |  |  |
| **d.** Family member 4 | |  | |  |  |  | |  |  |  |
| **e.** Family member 5 | |  | |  |  |  | |  |  |  |
| **f.** Family member 6 | |  | |  |  |  | |  |  |  |
| **g.** Family member 7 | |  | |  |  |  | |  |  |  |
| **h.** Family member 8 | |  | |  |  |  | |  |  |  |
| **i.** Family member 9 | |  | |  |  |  | |  |  |  |
| **j.** Family member 10 | |  | |  |  |  | |  |  |  |

3821139186

### Section 3: Your Use of Genetic and Screening Technologies

In this section, you will be asked about your previous use of genetic and screening technologies for thalassaemia.

**13. Are you, or your partner, currently pregnant or trying to get pregnant?**

Yes

No

Prefer not to say

| **14. In your current, or previous pregnancies, have you, or your partner, ever used any of the following technologies? Please tick all that apply. Please see**  **‘Glossary’ at the end of the survey for further explanation of these technologies. (Please go on to Section 4 if you, or your partner, have never been pregnant before)** |
| --- |
| CVS or Amniocentesis (diagnostic testing in pregnancy) for thalassaemia  CVS or Amniocentesis (diagnostic testing in pregnancy) for a condition other than thalassaemia  Pre-Implantation Genetic Diagnosis (PGD) (creation of embryos using IVF prior to testing) for thalassaemia  Pre-Implantation Genetic Diagnosis (PGD) (creation of embryos using IVF prior to testing) for a condition other than thalassaemia  Screening for Down's Syndrome (usually offered as a scan and/or blood test at around 12 weeks of pregnancy) Carrier Testing for thalassaemia  None of the above |

**15**

**. Have you, or your partner, ever undergone a pregnancy termination**

**(**

**abortion) due to thalassaemia?**

Yes

No

Prefer not to say

**15**

**a. If yes, please state how many terminations**

| **16. Have you, or your partner, ever undergone a pregnancy termination**  **(abortion) due to a prenatal diagnosis of a condition other than thalassaemia?** |
| --- |
| Yes No Prefer not to say |

### Section 4: Your Views on thalassaemia

In this section, you will be asked about your views about thalassaemia. You will be asked about how far you agree, or disagree, with, a list of statements. These statements were derived from interviews conducted with families living with thalassaemia and represent a wide range of views. This survey will measure how widespread these views are and how strongly they are held by others living with thalassaemia. Please note, there are no right and wrong answers to these questions. Everyone feels differently.

| **17. Please state how far you agree, or disagree with, the following statements about thalassaemia** | | | | | | |
| --- | --- | --- | --- | --- | --- | --- |
|  | **Views About THALASSAEMIA** | | | | | |
|  | **Strongly agree** | **Agree** | **Neither agree nor disagree** | **Disagree** | **Strongly disagree** | **Don't know** |
| **a.** People with thalassaemia can have a good quality of life |  |  |  |  |  |  |

1832139186

|  | **Views About THALASSAEMIA** | | | | |  |
| --- | --- | --- | --- | --- | --- | --- |
|  | **Strongly agree** | **Agree** | **Neither agree nor disagree** | **Disagree** | **Strongly disagree** | **Don't know** |
| **b.** Having thalassaemia causes those people to suffer |  |  |  |  |  |  |
| **c**. People with thalassaemia and their families are well supported by wider society |  |  |  |  |  |  |
| **d.** Quality of life with thalassaemia varies greatly depending on severity |  |  |  |  |  |  |

### Section 5: Your Views on Screening for Thalassaemia

In this section, you will be asked about your views on screening for thalassaemia. You will be asked how far you agree or disagree with a list of statements about thalassaemia, derived from interviews with families living with thalassaemia. These statements represent a wide range of views. This survey will measure how widespread they are, and how strongly they are held by the wider population of families living with thalassaemia. Screening involves the identification of thalassaemia in the UK population either pre-conceptually or in the newborn period. However, it cannot accurately predict the severity of the condition.

Screening for thalassaemia could be done in different ways:

1. **Pre-conception genetic screening** would identify ‘carriers’ of a thalassaemia gene (people who could transmit thalassaemia to future generations) before they have children or whilst they are planning a pregnancy. This would enable prospective parents to be informed of their risk of having a child with thalassaemia before the baby is even conceived.
2. **Newborn genetic screening** would identify babies with thalassaemia shortly after their birth through a blood test, ‘the heel prick test’. While some babies with thalassaemia are already being identified though this test when it is being used to look for other disorders, there is no formal newborn screening programme currently in place in the UK.

You will be asked a general question about screening for thalassaemia, before being asked to agree, or disagree with statements relating to preconception genetic screening and newborn genetic screening outlined above.

Please be reminded that some people may find the topic of screening for thalassaemia distressing. Should the completion of this survey raise any issues for you which you would like support with, or further information on, please contact your GP or treating clinician.

2753139189

1. **These statements relate to pre-conception genetic screening. Please state how far you agree or disagree with them by ticking in the appropriate box. Pre-conception genetic screening would allow people the option to know their carrier status before conceiving a pregnancy, so that they could be made aware from the outset of any future child's chance of being born with thalassaemia.**

|  | **Pre-Conception Genetic Screening for THALASSAEMIA** | | | | | |
| --- | --- | --- | --- | --- | --- | --- |
|  | **Strongly agree** | **Agree** | **Neither agree nor disagree** | **Disagree** | **Strongly disagree** | **Don't know** |
| **a.** Identifying carriers of thalassaemia before a pregnancy is conceived will affect people’s choice of reproductive partner (the person you choose to have a baby with) |  |  |  |  |  |  |
| **b.** It will be harder for thalassaemia carriers to get married and/or have children once their genetic status is  known about |  |  |  |  |  |  |
| **c.** Identifying carriers of thalassaemia in the general population will lead to carriers feeling stigmatised or different |  |  |  |  |  |  |
| **d**. Identifying carriers of thalassaemia before a pregnancy is established is a good thing, as it will reduce the number of terminations as parents will be aware of the chances beforehand |  |  |  |  |  |  |
| **e**. Identifying carriers of thalassaemia in the general population will increase awareness of thalassaemia as a condition |  |  |  |  |  |  |
| **f.** People from the general population won't be interested in finding out their carrier status as they won't think it's relevant to them |  |  |  |  |  |  |
| **g.** Pre-conception genetic screening is a form of ‘social engineering’ (a way of controlling the genetic make-up of the population) |  |  |  |  |  |  |
| **h.** I would support a pre-conception genetic screening programme for thalassaemia |  |  |  |  |  |  |

1. **These statements relate to newborn screening for thalassaemia. Pleasestate how far you agree or disagree with them.**

**This type of screening would identify babies affected by thalassaemia shortly after birth through a blood test. It might also identify some carriers of thalassaemia.**

|  | **Newborn Genetic Screening for THALASSAEMIA** | | | | | |
| --- | --- | --- | --- | --- | --- | --- |
|  | **Strongly agree** | **Agree** | **Neither agree nor disagree** | **Disagree** | **Strongly disagree** | **Don't know** |
| **a.** Identifying thalassaemia at birth would lead to better support and health care for the child and their family |  |  |  |  |  |  |
| **b.** Identifying thalassaemia at birth would extend the life expectancy of a child with thalassaemia |  |  |  |  |  |  |

0349139188

|  | **Newborn Genetic Screening for THALASSAEMIA** | | | | | |
| --- | --- | --- | --- | --- | --- | --- |
|  | **Strongly agree** | **Agree** | **Neither agree nor disagree** | **Disagree** | **Strongly disagree** | **Don't know** |
| **c.** Identifying thalassaemia before a child develops any symptoms prevents the child and their family from enjoying life whilst they are still symptom-free |  |  |  |  |  |  |
| **d**. Identifying thalassaemia at birth would help research into cure by enabling more children to be enrolled into clinical trials early on |  |  |  |  |  |  |
| **e**. Identifying thalassaemia at birth would interfere with the early bonding process between parent and child |  |  |  |  |  |  |
| **f.** Diagnosing thalassaemia at birth would make the diagnosis easier for parents to accept |  |  |  |  |  |  |
| **g.** Identifying thalassaemia at birth would spare parents the difficulties associated with finding a diagnosis for the child later on |  |  |  |  |  |  |
| **h.** Even though parents would not know for sure how severely affected their newborn baby will be, it's still better that they know about the thalassaemia straight away |  |  |  |  |  |  |
| **i.** Identifying thalassaemia at birth is  important as it would enable parents to make informed decisions about any future pregnancies |  |  |  |  |  |  |
| **j.** I would support a newborn genetic screening programme for thalassaemia |  |  |  |  |  |  |

### Section 6: Your Wider Views on Termination of Pregnancy (Abortion)

In this section, you will be asked about your wider views on pregnancy termination

(abortion). The question in this section is replicated from a 2003 survey, 'Views and Decisions about Prenatal Screening', which was given to pregnant women undergoing screening for Down's Syndrome. This question is being asked to explore whether families living with thalassaemia view termination of pregnancy (abortion) differently to women from the general population in the circumstances listed below.

**20. These statements relate to attitudes towards termination of pregnancy**

**(abortion). Please state whether you personally agree with a woman having a termination if...**

|  | **Wider Views on Termination of Pregnancy** | | | | | |
| --- | --- | --- | --- | --- | --- | --- |
|  | **Strongly agree** | **Agree** | **Neither agree nor disagree** | **Disagree** | **Strongly disagree** | **Don't know** |
| **a.** The family has a low income and cannot afford any more children |  |  |  |  |  |  |
| **b.** The woman decides she does not wish to have a child |  |  |  |  |  |  |
| **c.** The child is certain to have a serious mental disability and will  never live independently |  |  |  |  |  |  |
| **d.** The child is certain to have a serious physical disability and will never live independently |  |  |  |  |  |  |

3825139180

|  | **Wider Views on Termination of Pregnancy** | | | | | |
| --- | --- | --- | --- | --- | --- | --- |
|  | **Strongly agree** | **Agree** | **Neither agree nor disagree** | **Disagree** | **Strongly disagree** | **Don't know** |
| **e.** The child would live in good health, but would be certain to die in his/her twenties or thirties |  |  |  |  |  |  |
| **f.** The child would be healthy but would never grow taller than an eight year old (between 124cm and  132cm on average) |  |  |  |  |  |  |

**Thank you!**

Thank you for completing the Thalassaemia Screening Survey (UK). Please return your survey in the pre-paid envelope, or to the following address:

**Thalassaemia Screening Survey (UK)**

**C/O Natasha Nakariakova,**

**ITS, CEDAR Bld,**

**Warwick University**

**Coventry**

**CV4 7AL**

Some people may find the topic of screening distressing. Should the completion of this survey raise any issues for you which you would like to discuss further, please contact your GP or treating clinician.

Please feel free to pass this survey on to your relatives and/or any friends/acquaintances you know to be affected by thalassaemia. However, please be aware that screening can be a sensitive topic, and the views of others may not be the same as yours.

You can contact us (screeningstudy@warwick.ac.uk) for extra paper copies or use the link to the online version: http://www.warwick.ac.uk/thalassaemiascreeningsurvey (active from 21.05.2018).

You can find out more about the research project by visiting:

www.warwick.ac.uk/imagining_futures

#### Glossary of Terms

**CVS/Amniocentesis** - CVS and amniocentesis are diagnostic procedures used in pregnancy which usually involve the insertion of a needle through the abdomen to remove samples to be genetically tested.

**Pre-Implantation Genetic Diagnosis** - refers to the creation of embryos using IVF procedures. The embryos can then be tested for genetic conditions, such as thalassaemia, before being transferred back to the mother's uterus.

**Screening for Down’s Syndrome** - Down's Syndrome is a chromosomal disorder caused by an extra copy of chromosome 21 which leads to varying degrees of learning difficulty. It is also known to be associated with particular health problems, including heart problems, reduced vision and hearing, as well as early-onset Alzheimer's Disease (a form of senile Dementia). Screening for Down's Syndrome is offered to all pregnant women in the UK and usually involves a blood test and/or a scan (to measure the foetus’ nuchal fold- the back of the neck) at around 12 weeks of pregnancy.

4939139187
